# Dual role of p21 in regulating apoptosis and mitotic integrity in response to doxorubicin in colon cancer cells

**DOI:** 10.1038/s41420-025-02416-w

**Published:** 2025-04-02

**Authors:** Heeyeon Kim, Haein Kim, Eunjung Jang, Young-Woo Eom, Gyesoon Yoon, Kyeong Sook Choi, Eunhee Kim

**Affiliations:** 1 Department of Biological Sciences, Ulsan National Institute of Science and Technology (UNIST), Ulsan, South Korea; 2 Cell Therapy and Tissue Engineering Center, Yonsei University Wonju College of Medicine, Wonju, Korea; 3 Department of Biochemistry, Ajou University School of Medicine, Suwon, South Korea

**Keywords:** Chemotherapy, Apoptosis

## Abstract

This study explores the multifaceted role of p21 in mediating cellular responses to DNA-damaging agents, with a focus on doxorubicin treatment in HCT116 colon carcinoma cells. We investigated how different doses of doxorubicin affect cells with varied p21 and p53 statuses, revealing distinct roles for p21 depending on the drug dosage. At high doses (HD), p21 is more critical than p53 in mediating apoptosis, whereas at low doses (LD), p21 is essential for preventing mitotic defects and multinucleation. Notably, reintroducing p21 or pharmacologically inhibiting CDK1 reduced multinucleation. The absence of p21 upon LD doxorubicin exposure led to aberrant chromosome segregation, persistent DNA damage response (DDR) activation, and increased non-homologous end-joining (NHEJ) activity, resulting in unrepaired DNA accumulation and multinucleation. Additionally, mitotic defects in p21-deficient cells were associated with mislocalization of key mitotic regulators, Aurora B and mitotic kinesin-like protein 1 (MKLP1), exacerbating defective mitosis. In summary, p21 functions as a dual regulator in response to DNA damage, promoting apoptosis at HD and preventing mitotic failure at LD. These insights have significant implications for cancer therapy, highlighting the potential of targeting the p21 to enhance treatment efficacy.

## Introduction

Cellular responses to DNA damage are crucial for maintaining genomic stability and preventing tumorigenesis. Cells rely on key regulatory proteins, including p53 and p21, to direct responses to DNA damage, deciding whether to halt cell division, undergo repair, or initiate apoptosis based on the severity of the damage [1]. p53, often referred to as the “guardian of the genome”, and p21, a downstream effector of p53, are central to these processes, as they modulate both cell cycle arrest and apoptosis pathways [2, 3]. Doxorubicin, a chemotherapeutic agent known to induce DNA damage, has been widely utilized to study the roles of these proteins across diverse cellular contexts and varying levels of DNA damage [4]. In prior work, we demonstrated that doxorubicin induces two distinct modes of cell death in hepatocellular carcinoma cells with mutant p53, depending on the dose: high doses lead to apoptosis, whereas low doses result in cell death through mitotic catastrophe with a senescence-like phenotype [5]. However, the specific effects of doxorubicin dosage on p53- and p21-mediated cellular outcomes, especially in terms of cell cycle progression and apoptosis, remains inadequately understood.

p21, a cyclin-dependent kinase (CDK) inhibitor, primarily regulates the cell cycle by enforcing G1 and G2/M phase arrests in response to DNA damage [6]. Recent evidence suggests that p21’s functions extend beyond cell cycle regulation, potentially contributing to mitotic integrity and DNA repair [7–9]. However, delineating the specific contributions of p21 to genomic stability during sublethal DNA damage and its role in apoptosis at higher DNA damage levels remain challenging due to the complexity of its interactions with various molecular pathways. This study aims to clarify the dose-dependent roles of p21 and p53 in DNA damage response, focusing on how variations in their expression influence cellular outcomes, such as multinucleation, apoptosis, and the activation of specific repair pathways.

In this work, we used human colon carcinoma HCT116 cells with defined p53 and p21 statuses to investigate the cellular consequences of low and high doses of doxorubicin. Our findings reveal a distinct bifunctional role of p21: at low doses of doxorubicin, p21 is pivotal in maintaining mitotic progression and preventing multinucleation, while at high doses, it is essential for apoptosis induction. Additionally, we found that p21-deficient cells exhibit altered DNA damage responses and an increased reliance on error-prone repair pathways under mild DNA damage, leading to genomic instability. These results underscore p21’s importance in modulating dose-dependent cellular outcomes and highlight its potential as a therapeutic target for enhancing the efficacy of DNA-damaging agents in cancer treatment.

## Results

### Differential doses of doxorubicin elicit distinct cellular outcomes in HCT116 cells with varying 53 and p21 statuses

Both p53 and p21 are central regulators of the DNA damage response, particularly for cell cycle arrest and apoptosis [10–13]. To understand their contributions in cellular responses to DNA-damaging anticancer drugs, we treated isogenic human colon carcinoma HCT 116 cells with varying doses of doxorubicin, analyzing wild-type (WT), p53−/−, and p21−/− cell lines.

**Fig. 1:**
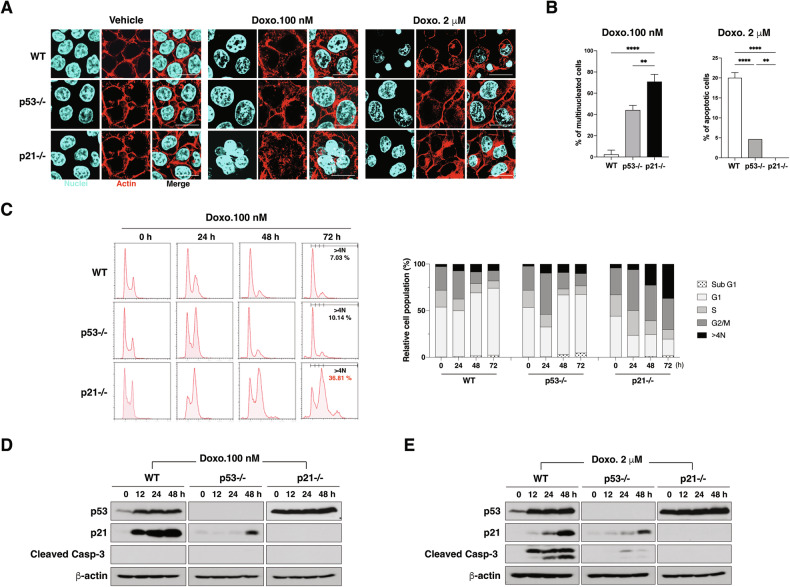
Effects of varying doxorubicin doses on cellular outcomes in HCT116 cells with different p53 and p21 statuses. **A** Immunofluorescence images of HCT116 WT, p53−/−, and p21−/− cells treated with 100 nM or 2 μM doxorubicin for 36 h, showing nuclear morphology (DAPI, cyan) and actin cytoskeleton (phalloidin, red). Scale bar = 20 μm. **B** Quantification of multinucleated (left) and apoptotic cells (right) in WT, p53−/−, and p21−/− cells treated with 100 nM or 2 µM doxorubicin (*n* = 30 cells per line). Data are presented as mean ± SD, **p < 0.01, ****p < 0.0001. **C** Flow cytometry of cell cycle distribution (sub G1, G1, S, G2/M and > 4 N) over time (0, 24, 48, and 72 h) in WT, p53−/−, and p21−/− cells treated with 100 nM doxorubicin, with quantification of cells with > 4 N DNA content. Histograms (left) and quantitation (right) are shown. **D**, **E** Western blot analysis of p53, p21, and cleaved caspase-3 expression in WT, p53−/−, and p21−/− cells treated with 100 nM (D) or 2 µM (E) doxorubicin. β-actin was used as a loading control.

At low doses (LD, 0.1 μM), doxorubicin induced differential effects based on cellular p53 and p21 status. Fluorescence microscopy revealed minimal changes in nuclear morphology in WT cells, while p53−/− cells showed moderate nuclear enlargement. In contrast, p21−/− cells displayed pronounced mutinucleation (Fig. 1A). Quantitative analysis indicated that multinucleated cells accounted for approximately 80% of the p21−/− cell population under LD doxorubicin treatment, underscoring p21’s role in maintaining genomic stability (Fig. 1B). Further investigation with flow cytometric analysis showed that at 24 h post-treatment with LD doxorubicin, all cell lines accumulated in the G2/M phase (Fig. 1C). By 48–72 h, WT and p53−/− cells transitioned to G1 arrest, while many p21−/− cells remained in the G2/M phase and displayed a progressive increase the population with DNA content > 4 N, indicative of mitotic failure. The subG1 population was minimal in all cells treated with LD doxorubicin (Fig. 1C). Consistent with these results, caspase-3 activation, a hallmark of apoptosis, was not observed in WT, p53−/−, or p21−/− cells, despite severe multinucleation in p21−/− cells (Fig. 1D). These results suggest that p21 is necessary for G1 arrest and for protecting mitotic defects under LD doxorubicin treatment.

At high doses (HD, 2 μM) doxorubicin treatment induced apoptosis in WT cells, characterized by chromatin condensation and fragmentation. However, p53−/− cells exhibited only mild nuclear enlargement, and p21−/− cells showed no apoptotic features, underscoring p21’s essential role in HD doxorubicin-induced apoptosis (Fig. 1A, B). Caspase-3 activation was robust in WT cells but minimal in p53−/− cells and absent in p21−/− cells, reinforcing p21’s critical role in facilitating apoptosis under HD DNA damage (Fig. 1E).

### p21’s role in regulating cellular responses to other DNA-damaging agents

To assess whether p21’s role is conserved across DNA-damaging agents, we treated cells with low-dose (5 μM) and high-dose (50 μM) etoposide. Low-dose etoposide induced multinucleation in p21−/− cells, whereas high-dose etoposide triggered apoptosis predominantly in WT cells, similar to LD and HD doxorubicin’s effects (Fig. S1A, B). These findings confirm p21’s role in preventing multinucleation at low doses and promoting apoptosis at high doses across DNA-damaging agents.

### p21 may enhance apoptosis by modulating the Noxa/Mcl-1 balance

**Fig. 2:**
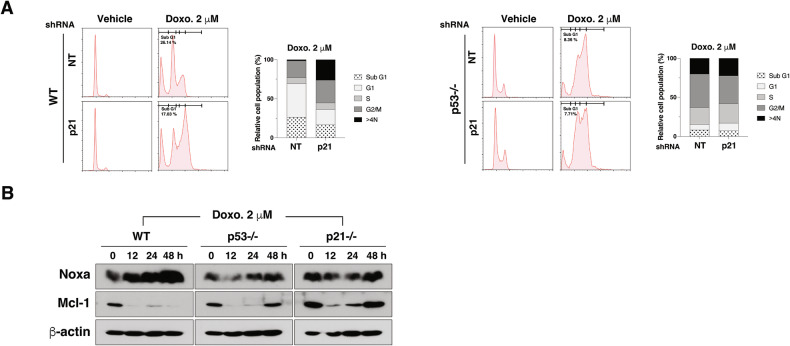
Role of p21 in high-dose doxorubicin-induced apoptosis. **A** Flow cytometry of cell cycle in HCT116 cells transduced with NT or p21 shRNA, treated with 2 µM doxorubicin for 48 h, showing subG1 cell population percentages. **B** Western blot analysis of Noxa and Mcl-1 expression in WT, p53−/−, and p21−/− cells treated with 2 µM doxorubicin. β-actin was used as a loading control.

To validate p21’s involvement in HD doxorubicin-induced apoptosis, we used shRNA to knock down of p21, resulting in a reduced sub-G1 population upon HD doxorubicin treatment (Fig. 2A). HD doxorubicin treatment upregulated the pro-apoptotic protein Noxa and downregulated the anti-apoptotic protein Mcl-1 in WT cells, but not in p53−/− and p21−/− cells (Fig. 2B). These results suggest that p21 may enhance HD doxorubicin-induced apoptosis by modulating the Noxa/Mcl-1 balance.

### p21 prevents LD doxorubicin-mediated multinucleation

**Fig. 3:**
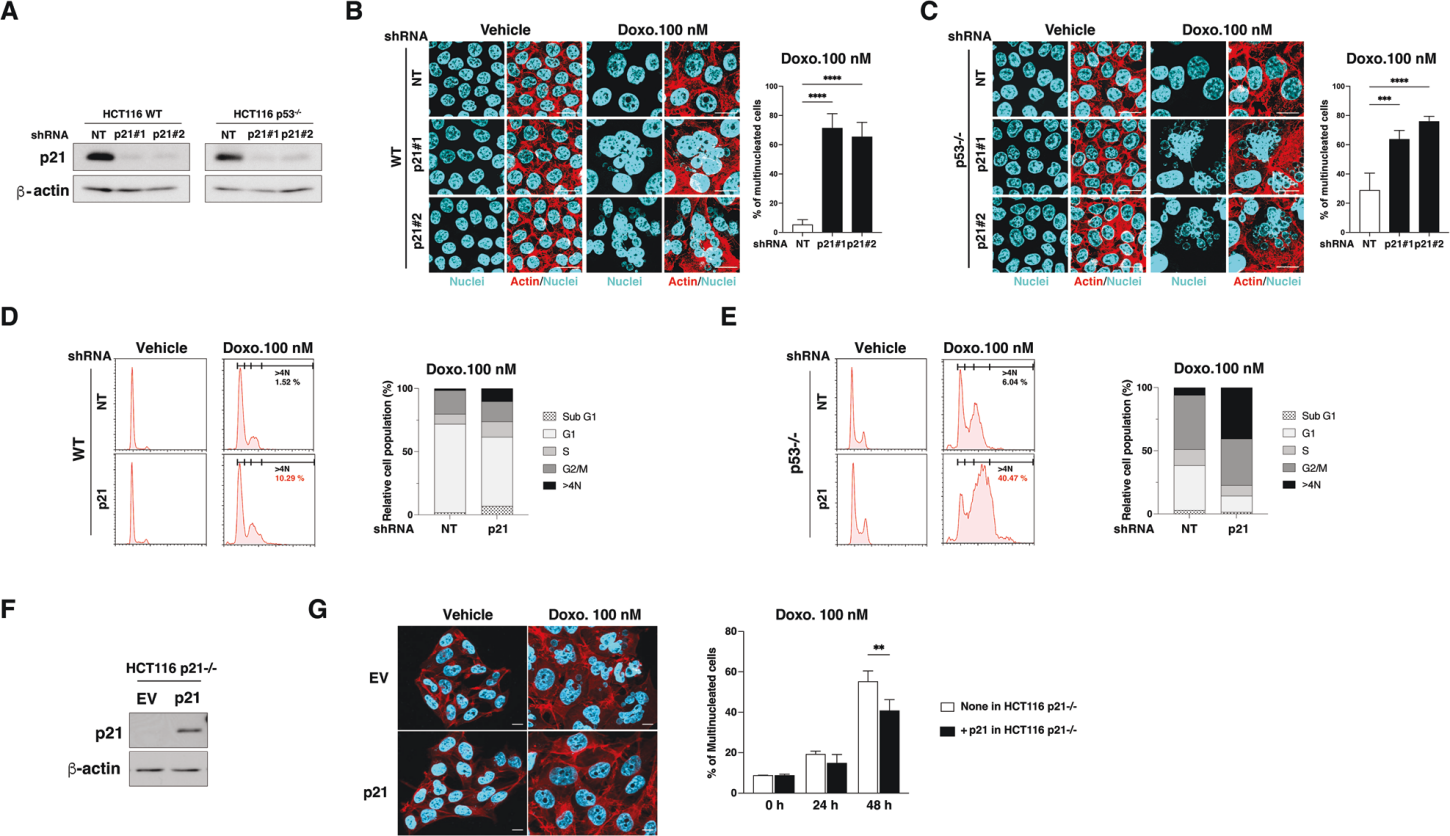
p21 is essential for maintaining genomic stability and normal mitotic progression. **A** Western blot analysis of p21 in HCT116 WT and p53−/− cells transduced with non-targeting (NT) or p21 shRNAs. β-actin was used as a loading control. **B**, **C** Fluorescence microscopy of nuclear morphology in HCT116 WT (**B**) and p53−/− (**C**) cells transduced with NT or p21-targeting shRNAs, treated with vehicle or 100 nM doxorubicin for 48 h. Nuclei and actin were stained with DAPI (cyan) and phalloidin (red), respectively. Scale bar = 20 µm. Quantitation of multinucleated cells is shown on the right (*n* = 50 cells per group). Data are mean ± SD, ***p < 0.001, ****p < 0.0001. **D**, **E** Flow cytometry analysis of cell cycle phases (Sub G1, G1, S, G2/M and > 4 N) in WT (D) and p53−/− (**E**) cells transduced with NT or p21 shRNA, treated with vehicle or 100 nM doxorubicin for 48 h, including quantification of cells with > 4 N DNA content. **F** Western blot showing p21 levels in HCT116 p21−/− cells transfected with empty vector (EV) or p21, with β-actin as a loading control. **G** Fluorescence microscopy of nuclear morphology (cyan) and actin (red) in HCT116 p21−/− cells transfected with EV or p21 after treatment with vehicle or 100 nM doxorubicin for 48 h. Scale bar = 10 μm. Quantitation of multinucleated cells (%) following 24 and 48 h of treatment is shown on the right (*n* = 50 cells per group). Data are mean ± SD, **p < 0.01.

To explore p21’s role in LD doxorubicin-induced multinucleation, we knocked down p21 using shRNA in both WT and p53−/− cells, which led to increased multinucleation and a higher population with DNA content > 4 N (Fig. 3A–E). Reintroducing p21 in p21−/− cells reduced multinucleation under LD doxorubicin treatment (Fig. 3F, G), confirming p21’s essential function in mitotic fidelity under LD conditions.

### p21 is required for proper mitotic progression in response to low-dose doxorubicin

**Fig. 4:**
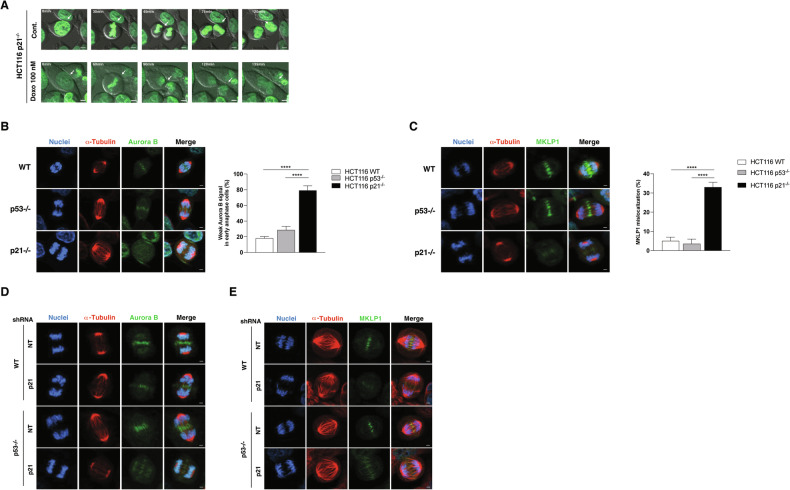
Mitotic defects in p21-deficient cells following low-dose doxorubicin treatment. **A** Live-cell imaging of HCT116 p21−/− cells expressing GFP-H2B, treated with 100 nM doxorubicin for 48 h. Images were captured every 15 min over 9 h. Scale bar = 5 μm. **B**, **C** Immunofluorescence showing the localization of Aurora B (**B**) and MKLP1 (**C**) in WT, p53−/−, and p21−/− cells synchronized to early anaphase by a thymidine-RO-3306 block. Hoechst 33342 (blue), α-tubulin (red), and Aurora B or MKLP1 (green) are shown. Quantification of cells with weak Aurora B (**B**) and MKLP1 (**C**) signals in early anaphase (*n* = 25 cells per group, repeated 4 times). Data are mean ± SD, ****p < 0.0001. **D**, **E** Immunofluorescence showing Aurora B (**D**) and MKLP1 (**E**) localization in WT and p53−/− cells transduced with NT or p21 shRNA.

To further investigate the mechanisms underlying multinucleation in p21−/− cells treated with LD doxorubicin, we performed time-lapse imaging. The results revealed several mitotic defects, including chromosome misalignment, lagging chromosomes, unequal chromosome segregation, and eventual cell fusion—all leading to failed cytokinesis and multinucleation (Fig. 4A). Additionally, we observed delayed mitotic entry and prolonged cytokinesis in these cells, indicating mitotic dysfunction. We examined key mitotic regulators, Aurora B [14–16] and MKLP1 [17–19], essential for chromosome segregation and cytokinesis, respectively. In p21−/− cells, both Aurora B and MKLP1 showed weak and diffuse expression, lacking proper central spindle localization, which is critical for successful cytokinesis (Fig. 4B, C). This mislocalization was also observed in WT and p53−/− cells following p21 knockdown (Fig. 4D, E). These findings indicate that the absence of p21 disrupts the Aurora B- and MKLP1-mediated mitotic checkpoint, leading to mitotic defects that contribute to multinucleation. Thus, p21 deficiency in LD doxorubicin-treated cells compromises both mitotic integrity and cytokinesis, resulting in a profound disruption of genomic stability.

### Persistent DNA damage response in p21-deficient cells treated with low-dose doxorubicin

**Fig. 5:**
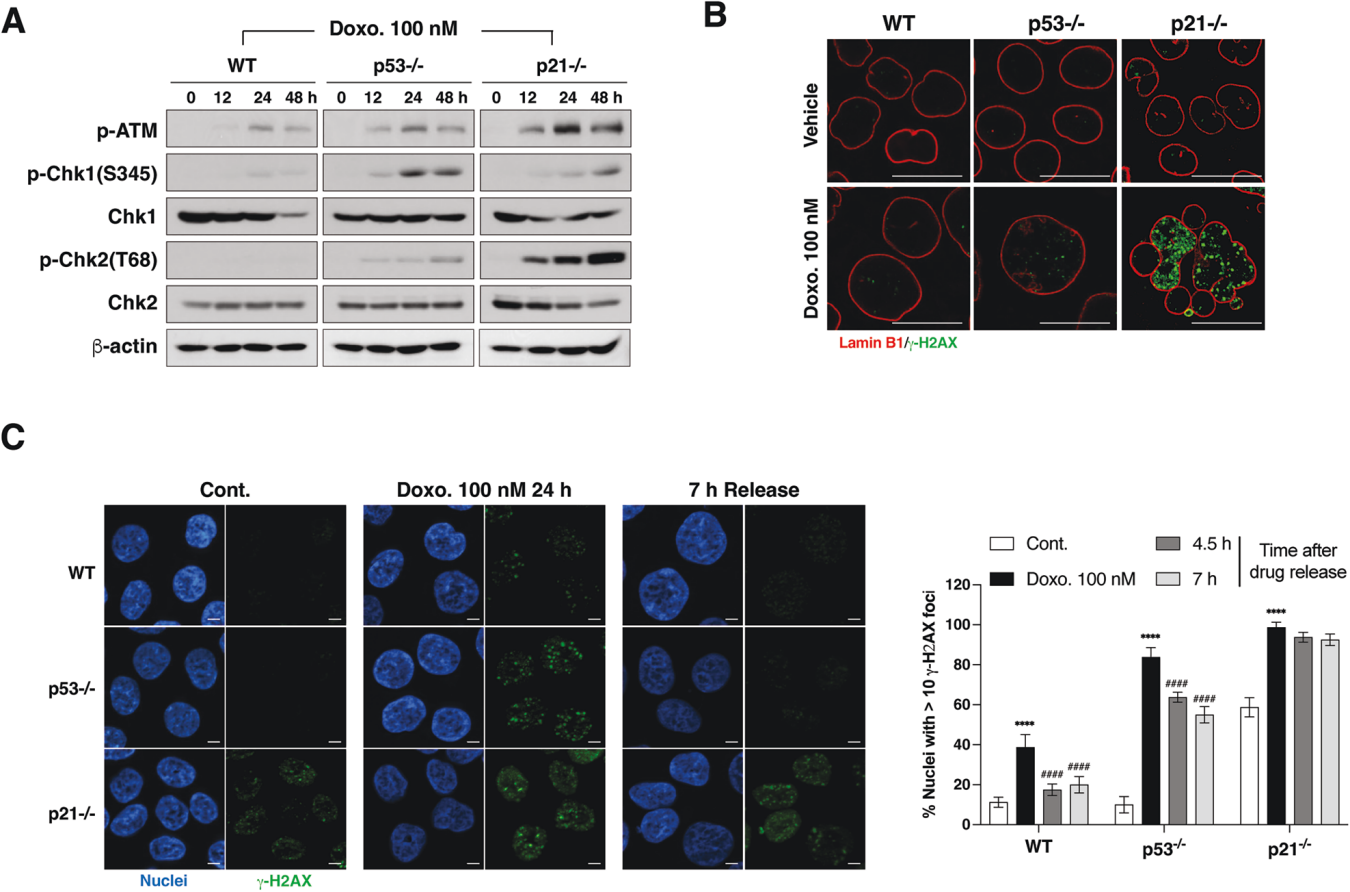
Impaired DNA damage repair in p21-deficient cells after low-dose doxorubicin treatment. **A** Western blot analysis of phosphorylated ATM (p-ATM), p-Chk1, Chk1, p-Chk2, and Chk2 in WT, p53−/−, and p21−/− cells treated with 100 nM doxorubicin for various time points. β-actin was used as a loading control. **B** Fluorescence microscopy of Lamin B1 (red) and γ-H2AX foci (green) in WT, p53−/−, and p21−/− cells treated with 100 nM doxorubicin for 48 h. Scale Bar = 20 μm. **C** Fluorescence microscopy showing nuclei (blue) and γ-H2AX foci (green) in HCT116 WT, p53−/−_,_ and p21−/− cells treated with 100 nM doxorubicin for 24 h and then released into fresh media for various time points. Scale bar = 20 μm. Quantification of nuclei with >10 γ-H2AX foci (*n* = 20 cells per group, repeated 4 times). Data are mean ± SD ****p < 0.0001.

We next investigated whether the distinct cellular outcomes in p53- and p21-deficient cells treated with LD doxorubicin were linked to differences in the DNA damage response (DDR). In p21−/− cells, ATM and Chk2 activation was significantly stronger compared to WT or p53−/− cells after LD doxorubicin treatment (Fig. 5A). The DNA damage marker γ-H2AX was dramatically elevated in p21−/− cells treated for 48 h, while WT and p53−/− cells showed minimal expression (Fig. 5B). This amplified DDR in p21−/− cells, characterized by persistent γ-H2AX signals even 7 h after doxorubicin washed out, but not in WT and p53−/− cells (Fig. 5C), suggests sustained DNA damage in these cells, indicating p21’s role in facilitating DNA repair. In contrast, efficiently resolved γ-H2AX signals, indicating more efficient DNA repair during LD doxorubicin-induced cell cycle arrest.

### Activation of error-prone DNA repair pathways in p21-deficient cells treated with low-dose doxorubicin

**Fig. 6:**
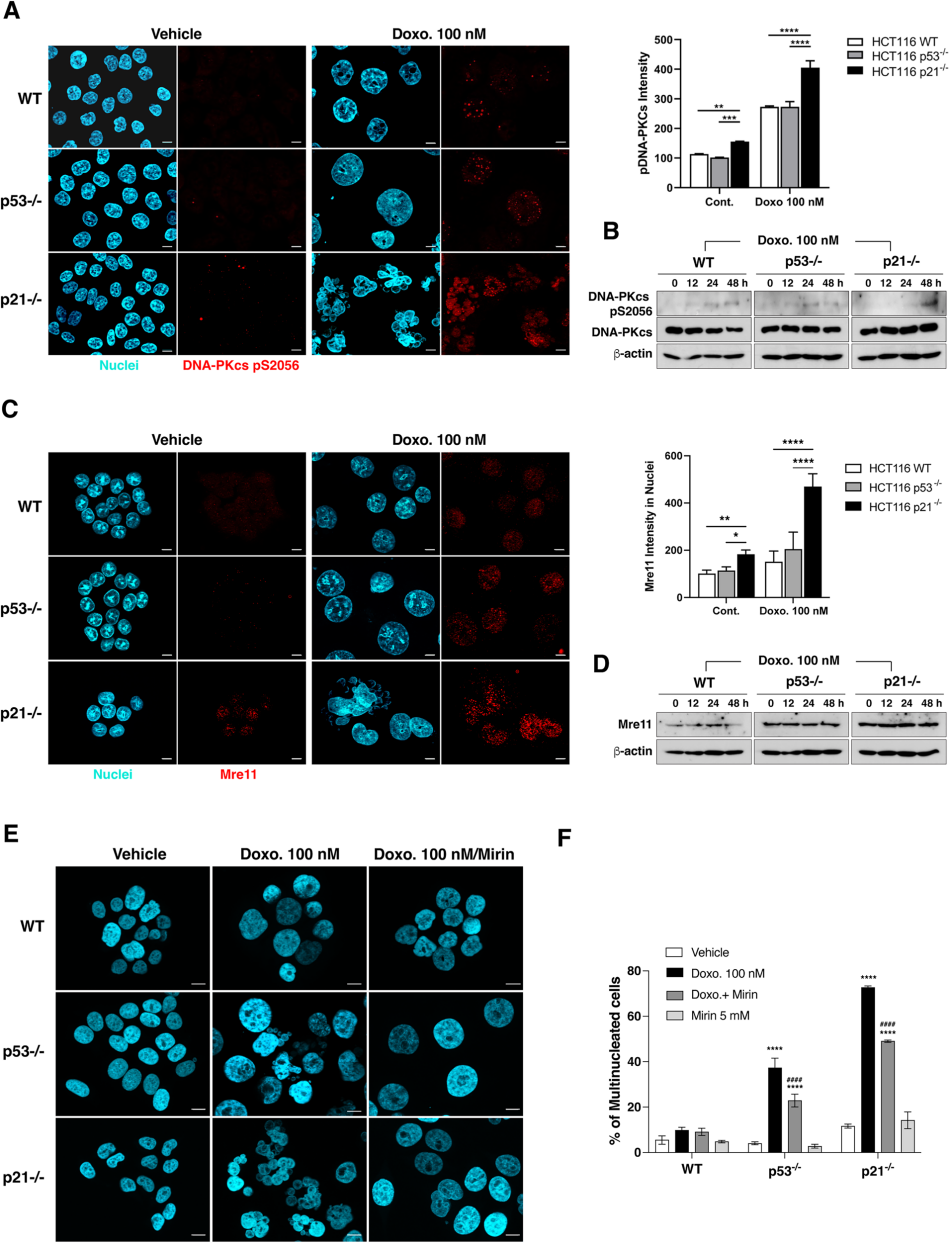
Activation of error-prone DNA repair pathways in p21-deficient cells treated with low-dose doxorubicin. **A**, **C** Immunofluorescence showing DNA-PKcs phosphorylation (**A**) or Mre11 (**C**) (red) in WT, p53−/−, and p21−/− cells treated with vehicle or 100 nM doxorubicin for 48 h. Nuclei were stained with Hoechst 33342 (cyan). Quantification of DNA-PKCs pS2056 (**A**) or Mre11 (**C**) signal intensity is shown (*n* = 16 cells per group, repeated 3 times). Scale bar = 10 μm. Data are mean ± SD **p < 0.01, ***p < 0.001, ****p < 0.0001. **B**, **D** Western blot of DNA-PKcs pS2056, DNA-PKcs (**B**) or Mre11 (**D**) in WT, p53−/−, and p21−/− cells treated with 100 nM doxorubicin for the indicated time points. β-actin was used as a loading control. **E** Fluorescence microscopy showing nuclear morphology (cyan) in WT, p53−/−, and p21−/− cells treated with vehicle, Mirin, or doxorubicin + Mirin for 48 h. Scale bar = 10 μm. **F** Quantification of multinucleated cells (%) following treatment as described in panel (**E**). data are mean ± SD, ****p < 0.0001 vs. vehicle, ^####^*p* < 0.0001 vs. Doxorubicin.

Given the lack of apoptosis in p21-deficient cells treated with LD doxorubicin, despite persistent DNA damage response (DDR) and nuclear aberrations (Figs. 1 and 4), we assessed error-prone DNA repair pathways. Elevated levels of phosphorylated DNA-dependent protein kinase catalytic subunit (DNA-PKcs) at serine 2056 (pS2056), a marker of non-homologous end-joining (NHEJ) [20, 21], were detected in untreated p21−/− cells. These levels were further increased following LD doxorubicin (Fig. 6A, B). Additionally, meiotic recombination 11 (Mre11), a key component of the MRN complex involved in DNA end processing for NHEJ [22], showed increased foci formation and protein levels in p21−/− cells treated with LD doxorubicin (Fig. 6C, D). Pretreatment with the Mre11 inhibitor mirin significantly reduced multinucleation in both p21−/− and p53−/− cells (Fig. 6E, F), suggesting that error-prone NHEJ activation in the absence of p21 leads to genomic instability under mild DNA damage conditions.

## Discussion

Our study provides new insights into p21’s dual, dose-dependent role in cellular responses to DNA damage, especially in doxorubicin treatment contexts. We demonstrate that p21 governs apoptosis at high doses while preserving mitotic integrity at low doses, highlighting its essential function in genomic stability and cell death regulation.

At high doses (HD) of doxorubicin, p21 facilitates apoptosis independent of p53, as evidenced by caspase activation failure in p21-deficient cells. These findings suggest that targeting the p21 pathway could enhance apoptotic responses to DNA-damaging agents in cancer therapy. At low doses (LD) of doxorubicin, p21 is crucial for preventing mitotic defects and multinucleation. The mitotic abnormalities we observed in p21-deficient cells—chromosome misalignment, lagging chromosomes, and failed cytokinesis—corroborate previous findings on p21’s role in protecting against genomic instability by preventing premature mitotic entry [7]. p21 may prevent multinucleation, particularly through CDK1 inhibition, which is crucial at the G2/M transition. Supporting this idea, pretreatment with the selective CDC inhibitor RO-3306 significantly reduced LD doxorubicin-induced multinucleation in p21−/− cells (Fig. S2). This dysregulation, coupled with Aurora B and MKLP1 mislocalization compromises chromosome segregation, resulting in mitotic defects and genomic instability.

Previous studies by Mauro et al. showed that p21-deficient cells rely more on NHEJ to repair DNA double-strand breaks [23]. Our findings extend this by demonstrating that persistent activation of NHEJ in p21-deficient cells, combined with a sustained DDR, leads to increased multinucleation and genomic instability.

In summary, our study clarifies p21 dual role in DNA damage responses, showing that it prevents mitotic defects and maintains genomic integrity at low doses of doxorubicin, while promoting apoptosis at high doses. These findings underscore the therapeutic potential of targeting p21 pathways to enhance the efficacy of DNA-damaging agents in cancer therapy. Further studies should aim to further delineate the molecular mechanisms by which p21 coordinates these distinct roles in DNA damage response.

## Materials and methods

### Chemicals and antibodies

Doxorubicin and etoposide were purchased from Selleckchem (Houston, TX, USA). Mirin, RO-3306, thymidine, and nocodazole were obtained from Sigma-Aldrich (St. Louis, MO, USA). Texas red-X Phalloidin and Hoechst 33342 were sourced from Thermo Fisher Scientific (Waltham, MA, USA). The following antibodies were used: β-actin (sc-69879), p53 (sc-126), Lamin B1(sc-365214), α-tubulin (sc-5286), Noxa (sc-515840, and MKLP1 (sc-136473) (Santa Cruz Biotechnologies, Dallas, TX, USA); p21 (ab109520), phospho-ATM (S1981) (ab81292), and phospho-DNA PKcs (S2056) (ab18192)(Abcam, Cambridge, MA, USA); phospho-Chk1 (Ser345)(# 2348), Chk1 (#2360), phospho-Chk2 (Thr68)(# 2197), Chk2 (#6334), cleaved caspase-3 (#9664), phospho-Histone H2A.X (Ser139)(# 9718), Mcl-1 (#39224), and Aurora B (#3094) (Cell signaling technology, Danvers, MA, USA); Mre11(GTX70212) and α-tubulin (GTX112141) (Genetex, San Antonio, Texas, USA); DNA-PKcs (19983-1-AP) (Proteintech, Rosemont, IL, USA); HRP-conjugated rabbit IgG and mouse IgG (Jackson Immunoresearch, West Grove, PA, USA).

### Cell culture

HCT116 human colon cancer cell lines (WT, p53−/−, p21−/−) were generously provided by Dr. Bert Vogelstein (Johns Hopkins University). Cells were cultured in Dulbecco’s Modified Eagle’s Medium (DMEM) (Hyclone Laboratories, Logan, UT, USA) supplemented with 10% v/v fetal bovine serum (FBS) (Hyclone Laboratories, Logan, UT, USA) and 1% v/v penicillin-streptomycin (10,000 units penicillin and 10,000 μg streptomycin) (Gibco-BRL). Cells were maintained at 37 °C in a 5% CO_2_ atmosphere.

### Small hairpin RNA-mediated knockdown of p21

To knock down p21, HEK 293T cells were transfected with the plasmids containing non-targeting shRNA (SHC002V, Sigma Aldrich), p21-targeting shRNA (TR305469, Origene), pVSV-G, and psPAX2.0, using the CalPhos Mammalian Transfection Kit (Takara Bio, Kusatsu, Japan) following the manufacturer’s instructions. After 48 h of lentiviral production, filtered viral supernatants from HEK293T cultures were used to infect HCT116 WT and p53−/− cells. Western blotting was performed to confirm successful p21 knockdown.

### Plasmid transfection

The pcDNA3-HA-p21 plasmid (Addgene plasmid #78782; RRID) [24] was used for transfection. HCT116 p21−/− cells were seeded in a 24-well plate and transiently transfected with the plasmid using X-tremeGENE^TM^ HP DNA Transfection Reagent (Roche, Indianapolis, IN) according to the manufacturer’s instructions. After 48 h of transfection, cells were used for subsequent experiments.

### Western blotting

Total cell lysates were prepared using Pierce IP lysis buffer (87787, Thermo Fisher Scientific) supplemented with 1X Halt™ Protease and Phosphatase Inhibitor Cocktail (Cat# 78441, Thermo Fisher Scientific) following the manufacturer’s protocol. Protein concentrations were measured using the Bradford assay and absorbance was read at 595 nm using a microplate reader. Equal amounts of protein were denatured in SDS sample buffer at 95 °C for 5 min, resolved by SDS–PAGE, and transferred to Immobilon membranes (Bio-Rad Laboratories, Hercules, CA, USA). Membranes were blocked with 5% w/v skim milk for 1 h, incubated overnight with specific primary antibodies, and subsequently washed with Tris-Buffered Saline with 0.05% v/v Tween-20 (TBST). Horseradish peroxidase (HRP)-conjugated antibodies (anti-rabbit or anti-mouse) were applied for 1 h. Protein bands were visualized using ECL (GE Healthcare, Chicago, IL, USA), and β-actin was used as the loading control. Unprocessed scans of immunoblots are provided as supplementary material.

### Immunocytochemistry

Following treatments, cells were fixed with 4% v/v paraformaldehyde (Electron Microscopy Sciences, Hatfield, PA, USA) for 10 min in PBS at room temperature and blocked in 5% v/v normal goat serum (Thermo Fisher) for 30 min. Cells were incubated overnight at 4 °C with primary antibodies (anti-phospho-Histone H2A.X, cleaved caspase-3, Aurora B (Cell signaling Technology), phospho-DNA PKcs(S2056) (Abcam), Mre11 and α-tubulin (Genetex), Lamin B1, α-tubulin, and MKLP1 (Santa Cruze Biotechnologies) diluted in 5% v/v goat serum with 0.2% v/v TritonX-100 in PBS. Cells were washed and incubated with Alexa-conjugated secondary antibodies (488, 594, or 647; Thermo Fisher) for 1 h at room temperature. Cells were counterstained with 4′,6-diamidino-2-phenylindole (DAPI) (0.1 μg/ml) or Hoechst 33342 (0.1 μg/ml) for 10 min, mounted using ProLong Diamond Antifade Mountant (Thermo Fisher), and visualized using fluorescence microscopy (Carl Zeiss AxioObserver 7, Oberkochen, Germany) or confocal microscopy (Olympus, FV1000, Japan).

### Cell cycle analysis

After treatment, cells were harvested and fixed in 70% v/v cold ethanol for 30 min at 4 °C. Fixed cells were washed twice with 0.1% FACS buffer (0.1% w/v BSA in PBS) and stained with DAPI (10 μg/ml) for 15–30 min. Flow cytometry was performed using an LSRFortessa (BD Biosciences), and data were analyzed with FlowJo software (Ashland, OR, USA).

### Assessment of aneuploidy

Multinucleated cells were visualized using Hoechst 33342 and Texas red-X Phalloidin staining. Cells with more than one nucleus were considered multinucleated. Five frames per sample were counted, and data were averaged. Three independent experiments were conducted.

### Assessment of apoptosis

Apoptotic cells were identified based on nuclear morphological changes such as chromatin condensation and fragmentation, visualized using Hoechst 33342 and Texas red-X Phalloidin staining. Five frames per sample were counted, and data were averaged. Three independent experiments were performed.

### Cell synchronization

HCT116 WT, p53−/−, and p21−/− cells were synchronized at the G1/S boundary using a thymidine block (2 mM thymidine for 18 h). After being released into a fresh medium for 3 h, cells were arrested in G2 by treatment with (10 μM) RO-3306 for 7 h, followed by release for 95 min to reach anaphase.

### Time-lapse imaging

Time-lapse imaging of HCT116 WT, p53−/− and p21−/− cells expressing GFP-H2B was conducted 48 h after treatment with 100 nM doxorubicin. Imaging was performed using fluorescence microscopy (Carl Zeiss AxioObserver 7) at 37 °C in a 5% CO_2_ atmosphere for 9 h, with DIC and fluorescence images taken every 15 min.

### Statistical analysis

All experiments were repeated at least three times. All results are presented as the mean ± standard deviation (SD) unless otherwise stated. Statistical analysis was performed using GraphPad Prism 9 (GraphPad Software Inc, San Diego, CA, USA). The normality of data distribution was assessed using the Kolmogorov–Smirnov test, and homogeneity of variances was evaluated using Bartlett’s test. For data meeting the assumptions of normality and equal variance, statistical differences between groups were analyzed using one-way or multi-way analysis of variance (ANOVA), followed by Bonferroni’s multiple comparison test for post hoc analysis. For all tests, *p* < 0.05 was considered significant (*p < 0.05, **p < 0.01, ***p < 0.001, and ****p < 0.0001).

## Supplementary information

Supplementary Information

Full and uncropped western blots

## Data Availability

All data and information concerning this study will be provided upon request.

## References

[1] Vousden KH, Prives C. Blinded by the light: the growing complexity of p53. Cell. 2009;137:413–31. 19410540 10.1016/j.cell.2009.04.037

[2] Fisher DE. The p53 tumor suppressor: critical regulator of life & death in cancer. Apoptosis. 2001;6:7–15. 11321044 10.1023/a:1009659708549

[3] el-Deiry WS, Tokino T, Velculescu VE, Levy DB, Parsons R, Trent JM, et al. WAF1, a potential mediator of p53 tumor suppression. Cell. 1993;75:817–25. 8242752 10.1016/0092-8674(93)90500-p

[4] Gewirtz DA. A critical evaluation of the mechanisms of action proposed for the antitumor effects of the anthracycline antibiotics adriamycin and daunorubicin. Biochem Pharm. 1999;57:727–41. 10075079 10.1016/s0006-2952(98)00307-4

[5] Eom YW, Kim MA, Park SS, Goo MJ, Kwon HJ, Sohn S, et al. Two distinct modes of cell death induced by doxorubicin: apoptosis and cell death through mitotic catastrophe accompanied by senescence-like phenotype. Oncogene. 2005;24:4765–77. 15870702 10.1038/sj.onc.1208627

[6] Karimian A, Ahmadi Y, Yousefi B. Multiple functions of p21 in cell cycle, apoptosis and transcriptional regulation after DNA damage. DNA Repair (Amst). 2016;42:63–71. 27156098 10.1016/j.dnarep.2016.04.008

[7] Kreis NN, Sanhaji M, Rieger MA, Louwen F, Yuan J. p21Waf1/Cip1 deficiency causes multiple mitotic defects in tumor cells. Oncogene. 2014;33:5716–28. 24317508 10.1038/onc.2013.518

[8] Ticli G, Cazzalini O, Stivala LA, Prosperi E. Revisiting the function of p21(CDKN1A) in DNA repair: The influence of protein interactions and stability. Int J Mol Sci. 2022;23:7058. 35806061 10.3390/ijms23137058 PMC9267019

[9] Avkin S, Sevilya Z, Toube L, Geacintov N, Chaney SG, Oren M, et al. p53 and p21 regulate error-prone DNA repair to yield a lower mutation load. Mol Cell. 2006;22:407–13. 16678112 10.1016/j.molcel.2006.03.022

[10] Bunz F, Hwang PM, Torrance C, Waldman T, Zhang Y, Dillehay L, et al. Disruption of p53 in human cancer cells alters the responses to therapeutic agents. J Clin Investig. 1999;104:263–9. 10430607 10.1172/JCI6863 PMC408422

[11] Gartel AL, Tyner AL. Transcriptional regulation of the p21 (WAF1/CIP1) gene. Exp Cell Res. 1999;246:280–9. 9925742 10.1006/excr.1998.4319

[12] Giono LE, Manfredi JJ. The p53 tumor suppressor participates in multiple cell cycle checkpoints. J Cell Physiol. 2006;209:13–20. 16741928 10.1002/jcp.20689

[13] Vousden KH, Lu X. Live or let die: the cell’s response to p53. Nat Rev Cancer. 2002;2:594–604. 12154352 10.1038/nrc864

[14] Carmena M, Wheelock M, Funabiki H, Earnshaw WC. The chromosomal passenger complex (CPC): from easy rider to the godfather of mitosis. Nat Rev Mol Cell Biol. 2012;13:789–803. 23175282 10.1038/nrm3474 PMC3729939

[15] Fuller BG, Lampson MA, Foley EA, Rosasco-Nitcher S, Le KV, Tobelmann P, et al. Midzone activation of aurora B in anaphase produces an intracellular phosphorylation gradient. Nature. 2008;453:1132–6. 18463638 10.1038/nature06923 PMC2724008

[16] Liang C, Zhang Z, Chen Q, Yan H, Zhang M, Zhou L, et al. Centromere-localized Aurora B kinase is required for the fidelity of chromosome segregation. J Cell Biol. 2020;219:e201907092. 31868888 10.1083/jcb.201907092 PMC7041694

[17] Guse A, Mishima M, Glotzer M. Phosphorylation of ZEN-4/MKLP1 by aurora B regulates completion of cytokinesis. Curr Biol. 2005;15:778–86. 15854913 10.1016/j.cub.2005.03.041

[18] Papini D, Levasseur M, Higgins JM. The Aurora B gradient sustains kinetochore stability in anaphase. Cell Rep. 2021;37:109818. 34758321 10.1016/j.celrep.2021.109818 PMC8595645

[19] Zhu C, Bossy-Wetzel E, Jiang W. Recruitment of MKLP1 to the spindle midzone/midbody by INCENP is essential for midbody formation and completion of cytokinesis in human cells. Biochem J. 2005;389:373–81. 15796717 10.1042/BJ20050097 PMC1175114

[20] Kurimasa A, Kumano S, Boubnov NV, Story MD, Tung C-S, Peterson SR, et al. Requirement for the kinase activity of human DNA-dependent protein kinase catalytic subunit in DNA strand break rejoining. Mol Cell Biol. 1999;19:3877–84. 10207111 10.1128/mcb.19.5.3877 PMC84245

[21] Uematsu N, Weterings E, Yano K-I, Morotomi-Yano K, Jakob B, Taucher-Scholz G, et al. Autophosphorylation of DNA-PKCS regulates its dynamics at DNA double-strand breaks. J Cell Biol. 2007;177:219–29. 17438073 10.1083/jcb.200608077 PMC2064131

[22] Williams RS, Williams JS, Tainer JA. Mre11–Rad50–Nbs1 is a keystone complex connecting DNA repair machinery, double-strand break signaling, and the chromatin template. Biochem Cell Biol. 2007;85:509–20. 17713585 10.1139/O07-069

[23] Mauro M, Rego MA, Boisvert RA, Esashi F, Cavallo F, Jasin M, et al. p21 promotes error-free replication-coupled DNA double-strand break repair. Nucleic Acids Res. 2012;40:8348–60. 22735704 10.1093/nar/gks612 PMC3458556

[24] Lee M, Seo J, Choi D, Lee E, Ko A, Ha N, et al. Stabilization of p21 (Cip1/WAF1) following Tip60-dependent acetylation is required for p21-mediated DNA damage response. Cell Death Differ. 2013;20:620–9. 23238566 10.1038/cdd.2012.159 PMC3595487

