# Supplementary material for: Dual role of p21 in regulating apoptosis and mitotic integrity in response to doxorubicin in colon cancer cells
Source: Cell Death Discov. 2025 Apr 2;11:133. doi: 10.1038/s41420-025-02416-w (PMC11965415; doi:10.1038/s41420-025-02416-w)
Supplement: Supplementary file 1 — Supplementary Information [file 41420_2025_2416_MOESM1_ESM.docx]

**Supplementary Information**

**Dual role of p21 in regulating apoptosis and mitotic integrity in response to doxorubicin in colon cancer cells**

**Heeyeon Kim^1^, Haein Kim^1^, Eunjung Jang^1^, Young-Woo Eom^2^, Gyesoon Yoon^3^, Kyeong Sook Choi^3*^, Eunhee Kim^1*^**

^1^ Department of Biological Sciences, Ulsan National Institute of Science and Technology (UNIST), Ulsan, South Korea

^2^ Cell Therapy and Tissue Engineering Center, Yonsei University Wonju College of Medicine, Wonju, Korea

^3^ Department of Biochemistry, Ajou University School of Medicine, Suwon, South Korea

*Corresponding Author: Kyeong Sook Choi, Ph.D. E-mail: [kschoi@ajou.ac.kr](mailto:kschoi@ajou.ac.kr)

Eunhee Kim, Ph.D. E-mail: [ehkim@unist.ac.kr](mailto:ehkim@unist.ac.kr)

**Keywords:** p21, Doxorubicin, DNA damage response, Multinucleation, Anticancer therapy

**Subtitle:**

p21’s Dual Role in Apoptosis and Mitotic Integrity in Colon Cancer


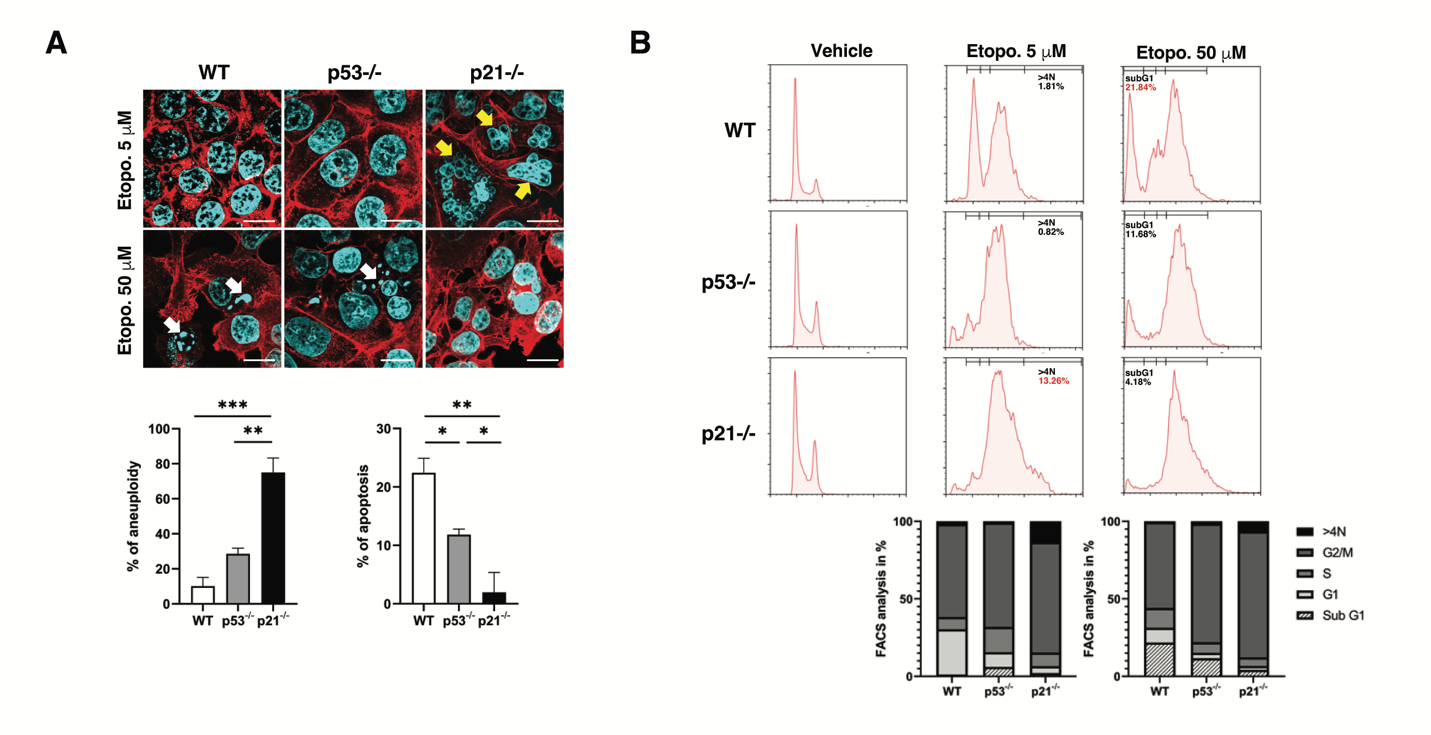


**Supplementary Fig. S1.** **Effects of varying doses of chemotherapeutic agents on nuclear morphology and DNA content in HCT116 cells with different p53 and p21 statuses.**

**A** HCT116 WT, p53^-/-^ and p21^-/-^ cells were treated with 5 μM (low-dose) and 50 μM (high-dose) etoposide for 48 hours. Cells were stained with DAPI (blue) and phalloidin (red). Yellow arrows indicate multinucleated cells; white arrows indicate apoptotic cells. Scale bar = 20 μm. **B** Flow cytometry of the cell cycle in HCT116 WT, p53-/- and p21-/- cells treated with 5 μM or 50 μM etoposide for 48 hours, showing representative histograms and quantification of cell cycle distributions.


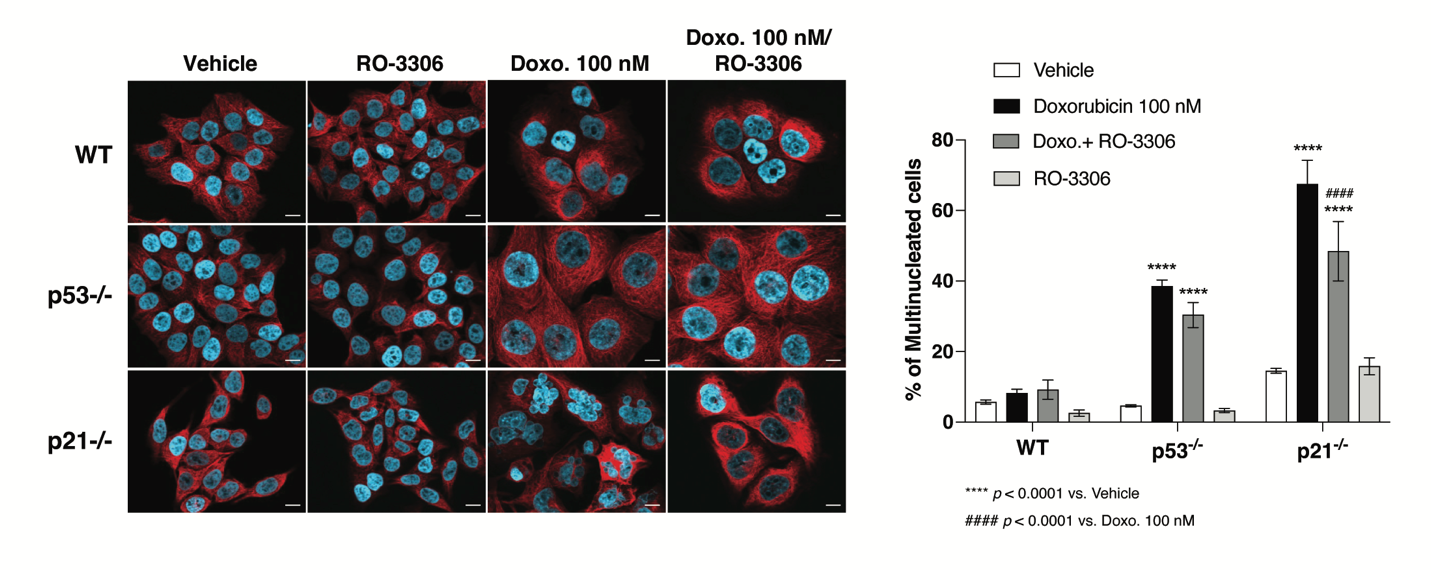


**Supplementary Fig. S2. CDK inhibition attenuates low-dose doxorubicin-induced multinucleation.**

Fluorescence microscopy of nuclear morphology (cyan) and actin (red) in WT, p53-/-, and p21-/- cells treated with vehicle, doxorubicin, doxorubicin + RO-3306, or RO-3306 alone. Scale bar = 10 μm. Quantification of multinucleated cells is shown on the right (n = 30 cells per group). Data are mean ± SD, *****p*< 0.0001 vs. vehicle; #*p*< 0.05, ####*p*< 0.0001 vs. doxorubicin.
