# Supplementary figures and images for: Dual role of p21 in regulating apoptosis and mitotic integrity in response to doxorubicin in colon cancer cells
Source: Cell Death Discov. 2025 Apr 2;11:133. doi: 10.1038/s41420-025-02416-w (PMC11965415; doi:10.1038/s41420-025-02416-w)

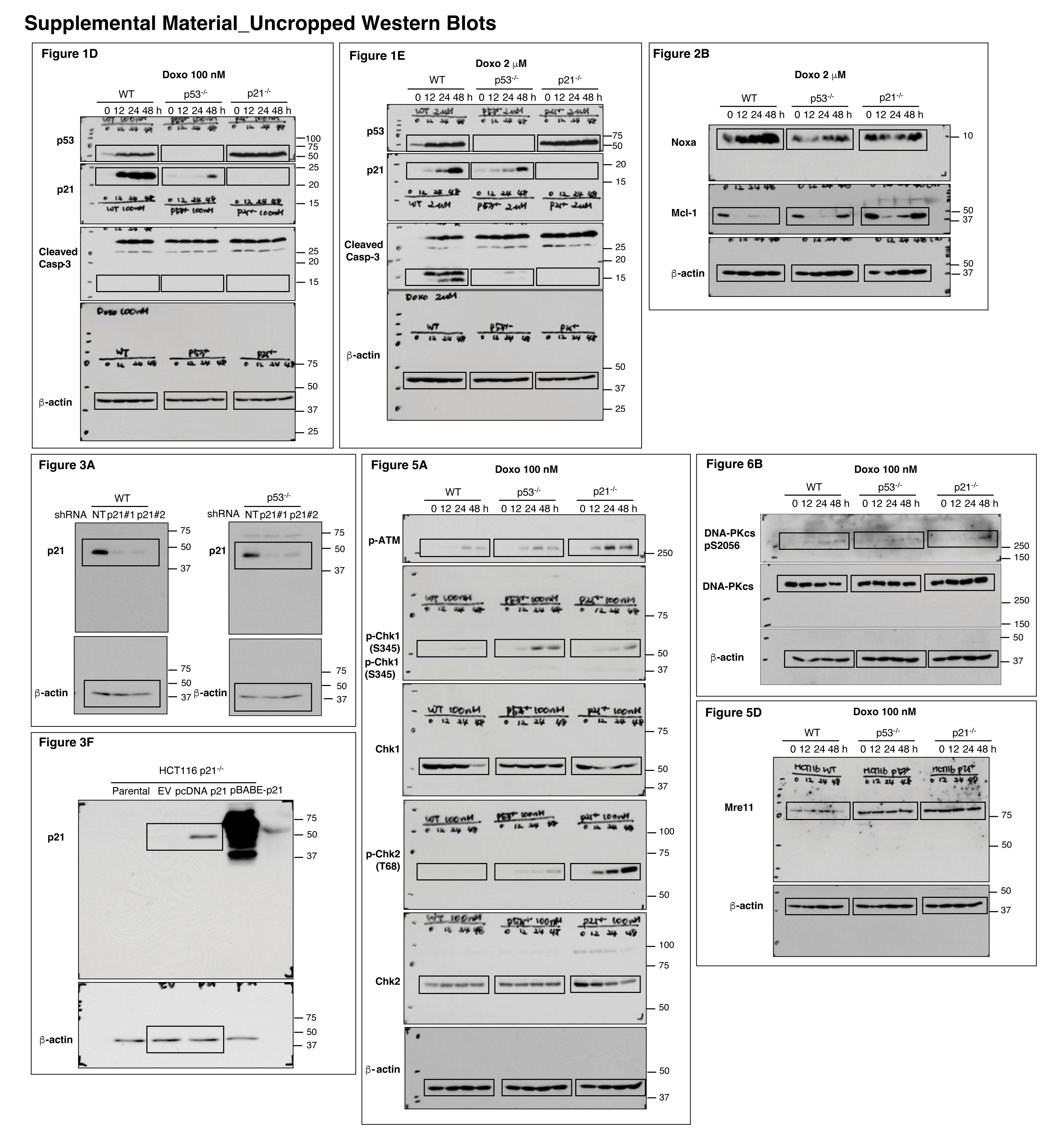

Supplement: Supplementary file 2 — Full and uncropped western blots [file 41420_2025_2416_MOESM2_ESM.tif]
